# Knowledge, attitude and practice towards kangaroo mother care among postnatal women in Ethiopia: Systematic review and meta-analysis

**DOI:** 10.1371/journal.pone.0265411

**Published:** 2022-05-06

**Authors:** Natnael Atnafu Gebeyehu, Kelemu Abebe Gelaw, Gedion Asnake Azeze, Biruk Adie Admass, Eyasu Alem Lake, Getachew Asmare Adela

**Affiliations:** 1 School of Midwifery, College of Health Science and Medicine, Wolaita Sodo University, Wolaita Sodo, Ethiopia; 2 Department of Anesthesia, College of Health Science and Medicine, University of Gondar, Gondar, Ethiopia; 3 School of Nursing, College of Health Science and Medicine, Wolaita Sodo University, Wolaita Sodo, Ethiopia; 4 School of Public Health, College of Health Science and Medicine, Wolaita Sodo University, Wolaita Sodo, Ethiopia; Debre Tabor University, ETHIOPIA

## Abstract

**Background:**

Kangaroo mother care is a key procedure in reducing neonatal mortality and morbidity associated with preterm birth. In Ethiopia, neonatal death remains a serious problem, and this study aims to determine the prevalence of the knowledge, attitudes and practice of kangaroo mother care among Ethiopia women.

**Methods:**

PubMed, Web of Science, Google Scholar, EMBASE and the Ethiopian University online library were searched. Data were extracted using Microsoft Excel and analysed using STATA statistical software (v. 11). Publication bias was checked by forest plot, Begg’s rank test and Egger’s regression test. To look for heterogeneity, I^2^ were computed and an overall estimated analysis carried out. Subgroup analysis was done by region, study setting, publication, gestational age, birth weight and component of kangaroo care. The Joanna Briggs Institute risk of bias assessment tool was used. We carried out a leave one out sensitivity analysis.

**Results:**

Out of 273 articles retrieved, 16 studies met the eligibility criteria and are thus included in this study. Those 16 studies had a total of 12,345 respondents who reported kangaroo mother practice, with five (comprising 1,232 participants combined) reporting that both knowledge and attitude were used to determine the overall estimation. The pooled estimates of good knowledge, positive attitude and poor practice of kangaroo mother care were found to be 64.62% (95% CI: 47.15%–82.09%; I^2^ = 97.8%), 61.55% (49.73%–73.38%; I^2^ = 94.8%) and 45.7% (95% CI: 37.23%–54.09%; I^2^ = 98.5%), respectively. This study is limited to postnatal women and does not take account their domestic partners or health providers.

**Conclusion:**

The findings revealed significant gaps in the knowledge, attitudes and practice of kangaroo mother care in Ethiopia when compared with other developing countries. Therefore, kangaroo mother care training to women, along with further studies on domestic partners and health providers.

## Introduction

Low birth weight and preterm birth represent the two major public health challenges during the neonatal period [1]. During this period, 36% of deaths occur on the day of birth, while 73% occur within the first week of life [2]. In addition, More than 80% of neonatal deaths occur in low birth weight neonates two-thirds of whom were are born prematurely [3]. Complications related to prematurity are the major cause of neonatal mortality [4]. Worldwide, 25 million babies are born a low birth weight annually while 15 million are born prematurely with 96% of these preterm babies occurring in developing countries [5, 6]. In Ethiopia, systematic review and meta-analysis studies have reported the pooled prevalence of preterm birth (to be 10.8%), and that of low birth weight (to be 17.3%) [7, 8].

Due to the prevalence of preterm births and low birth weight, as well as associated health care burden, comprehensive interventional procedures for primary prevention are required such as Kangaroo Mother Care [9]. According to the world health organization (WHO), kangaroo mother care involves an early, uninterrupted, and prolonged skin–to–skin contact between mother and the baby [10]. Although kangaroo mother care is recommended by World Health Organization, Baby Friendly Initiatives, United Nation International Children Emergency Fund, and American Academy of pediatrics, the prompt separation of the baby from the mother after birth remains a significant challenge [11–14].

Several studies have reported that kangaroo mother care to be a cost-effective intervention for reducing mortality and morbidity in preterm infants [15]. It has also been found to have a positive impact on maternal health in low, middle, and high-income countries [16–22]. In fact, studies have shown that kangaroo mother care reduced neonatal mortality [17, 23], sepsis [17, 23], hypothermia [17, 23], hypoglycemia [23], and length of hospital stay [17] when compared with conventional care approaches.

The findings of a number of systematic review studies have indicated that kangaroo mother care increased the success, initiation, and duration of breast feeding [24–29]. Moreover, it has also been found to improve maternal anxiety and stress [30], enhanced-cognitive and motor development [31], reduce the likelihood of hospital readmission [32, 33] and lowered the premature infant profile [34]. Another study reported that Kangaroo mother care improves the growth of low birth weight and preterm infants [35–41].

In Ethiopia, kangaroo mother care was first introduced in 1996 at Black Lion Hospital in Addis Ababa. Since then the service has been expanded to other health facilities and hospitals. In addition, the Federal Ministry of Health has integrated kangaroo mother care into National Strategy for Newborn and Child Survival, Health Sector Transformation Plan, and National Health Care Quality Strategy with the later focusing on ensuring that 80% of preterm babies to receive kangaroo mother care, although initiation currently remains low [42–44]. Moreover, the neonatal mortality rate in Ethiopia is 30 per 1000 live births, which means that the problem remains significant [45]. By the end of 2030, Ethiopia aims to have lowered the neonatal mortality rate to 12 deaths per 1000 live births [46]. To help achieve this target, Kangaroo mother care is expected to play a significant role in anticipating neonatal hypothermia [11].

The prevalence of kangaroo mother care has been determined to range from 1% in Tanzania [47] to 96% in Denmark [14]. In Ethiopia, knowledge of kangaroo mother care has been found to range from 35.5% to 82.53% [48–52], attitude from 50% to 82.53% [49–53] and practice from 23% to 83% across the nation [48–63]. Given these variations, there is no overall estimate of the prevalence of kangaroo mother care based on representative national data in Ethiopia. Therefore, the present study sought to determine the pooled prevalence of knowledge, Attitude, and practice regarding of kangaroo mother care among postnatal women in Ethiopia. The aim was to provide fundamental data for policymakers, clinicians, and other stakeholders in order to help develop an appropriate strategies and interventions for the control and management of low-birth weight and preterm birth using kangaroo mother care.

## Methods

### Searching strategy

To obtain the data required for this study, we performed manual searched and also searched on PubMed, Web of Science, Google Scholar, EMBASE, and various grey literature data bases (Addis Ababa University, Ethiopia). More specifically, we used Keywords, medical Subject headings (MeSH) terms and Emtree terms to conduct the search. We applied search terms independently and/or in combination using “OR”, “AND” or “NOT”. In EMBASE we used Boolean operators and Emtree terms (a controlled vocabulary or standard words used to make searching easier) to identify relevant articles, whereas in Web of science we used synonyms, Boolean operators, key words, and topics. In Google Scholar and when performing manual searches, we used a combination of the above mentioned key terms/phrases (i.e. “Knowledge of kangaroo mother care”, “attitude of kangaroo mother care”, “kangaroo mother care”, “low birth weight neonate”, “preterm neonate”, “newborn care practice”, “kangaroo mother”, “skin to skin care practice”, “skin to skincare”, “kangaroo mother care method, and Ethiopia) were used. The grey literature databases were searched via Ethiopian Universities Online repository library home. Our search strategy with regard to PubMed was as follows: ((((((((Knowledge [tw]) OR "Knowledge"[Mesh Terms]) AND (Attitude[tw] OR perception [tw])) OR "Attitude " [Mesh Terms]) AND Practice [tw]) OR "Practice" [Mesh Terms]) AND (Kangaroo mother care[tw] OR low birth weight neonate[tw]OR preterm neonate[tw] OR newborn care practice[tw])) OR ("Kangaroo mother care " [Mesh Terms] OR "Kangaroo mother " [Mesh Terms] OR "skin to skin care practice" [Mesh Terms] OR "skin to skin care"[Mesh Terms] OR" kangaroo mother care method" [Mesh Terms])) AND Ethiopia. The electronic literature search was performed from 30 May, 2021 to 30 June, 2021. All of the accessible studies that had been published in English from inception up to 30 June, 2021 were included in the present meta-analysis and systematic review.

In terms of reporting the findings of the literature search, The Preferred Reporting Items for Systematic Reviews and Meta-Analysis (PRISMA) guideline was used [64] (S1 File). This systematic review and meta-analysis study was not registered under Prospero, but we checked that any author has not registered it yet.

### Operational definitions

#### Knowledge

Those study participants who were responded to ≥50% of the knowledge-related questions were considered to have a good level of knowledge, while those who responded to less than 50% were considered to have a poor level of knowledge [48].

#### Attitude

Those study participants who answered ≥50% of the mean value of the on attitude-related questions were considered to exhibit a positive attitude towards kangaroo mother care whereas those who answered below the mean value of the attitude-related questions were considered to exhibit having a negative attitude.

#### Practice

Those study participants who responded to ≥50% of the practice-related questions were categorized as demonstrating good practice, whereas those respondents who answered <50% of the questions were considered to demonstrate poor practice.

### Eligibility criteria

All studies that reported the prevalence of knowledge, attitude and practice of kangaroo mother care, postnatal women as study participants, English language reporting, had full text available for search and took place in Ethiopia were included in this systematic review and meta-analysis. Those studies that reported duplicated sources, unrelated research, case reports, qualitative studies, and articles with no full text available attempts to contact the corresponding author via email were excluded this systematic review and meta-analysis.

### Study selection and data extraction

Three independent authors selected the candidate articles for the study, which were exported Endnote reference manager software to remove duplicate, and independently screened the titles and abstracts (NA, KA, and EA). Any disagreement was resolved through discussions lead by a third author. Data were extracted using a standardized data extraction format prepared in Microsoft Excel by four independent authors (NA, GA, BA and GA). Any disagreement that happened during extraction was resolved through discussion lead by the fourth author. The data automation tool was not used due to the absence of the paper form (manual data) in this study. The name of the first author, year of publication, study region, study setting, the prevalence of knowledge of kangaroo mother care, the attitudes towards kangaroo mother care, the practice of kangaroo mother care, sample size, gestational age, type of kangaroo mother care and birth weight were collected.

### Quality assessment

The two independent authors appraised the standard of the studies using the Joanna Briggs Institute (JBI) quality appraisal checklist [31]. Any disagreement was discussed and resolved by the authors. The critical analysis checklist has eight parameters with yes, no, unclear, and not an applicable options. The parameters involve the following questions:

1. Where were the criteria for inclusion in the sample clearly defined?
2. Were the study subjects and therefore the setting described in detail?
3. Was the exposure measured the result validly and reliably?
4. Were the main objective, standard criteria used for measurement of the event?
5. Were confounding factors identified?
6. Were strategies to affect confounding factors stated?
7. Were the results measured truly and dependably?, and (8) Was the statistical analysis suitable?. Studies were considered low risk when they scored 50% and above of the quality assessment indicators as reported in supplementary file (S2 File).

#### Risk of bias assessment

This systematic review and meta-analysis study used a risk of bias assessment tool developed by Hoy et al [65] consisting of ten items that assess four domains of bias, internal and external validity. The first four items (items1–4) evaluate the presence of selection bias, non- response bias and external validity. The other six items (items 5–10) assess the presence measuring the bias, analysis- related bias and internal validity. Therefore, if studies that received ‘yes’ for eight or more of the ten questions were classified as ‘low risk of bias.’ If studies that received ‘yes’ for six to seven of the ten questions were classified as ‘moderate risk’ whereas if studies that received ‘yes’ for five or fewer of the ten questions were classified as ‘high risk’ as reported in (S3 File).

### Statistical analysis

After data extraction was done using Microsoft Excel, the analysis was conducted by using STATA version 14 statistical software. Publication bias was checked by funnel plot and more objectively through Begg and Egger’s regression tests, with P< 0.05 considered to indicate potential publication bias. A trim and fill analysis was done to see the effect of publication bias. It adds studies to form the symmetrical distribution. The presence of between-study heterogeneity was checked by using Cochrane Q statistic. This heterogeneity between studies was quantified using I^2^, in which a values of 0, 25, 50, and 75% represented no, low, medium, and in-creased heterogeneity, respectively. A forest plot was used to visually assess the presence of heterogeneity, which presented at a high level random-effect model was used for analysis to estimate the overall prevalence of knowledge, attitude and practice of kangaroo mother care. Subgroup analysis was done by study setting, region, gestational age, birth weight, and type of kangaroo care practice. A sensitivity analysis was executed to see the effect of a single study on the overall prevalence of the meta-analysis estimate. The findings of the study were presented in the form of text, tables, and figures.

## Results

### Study selection

**Fig 1:**
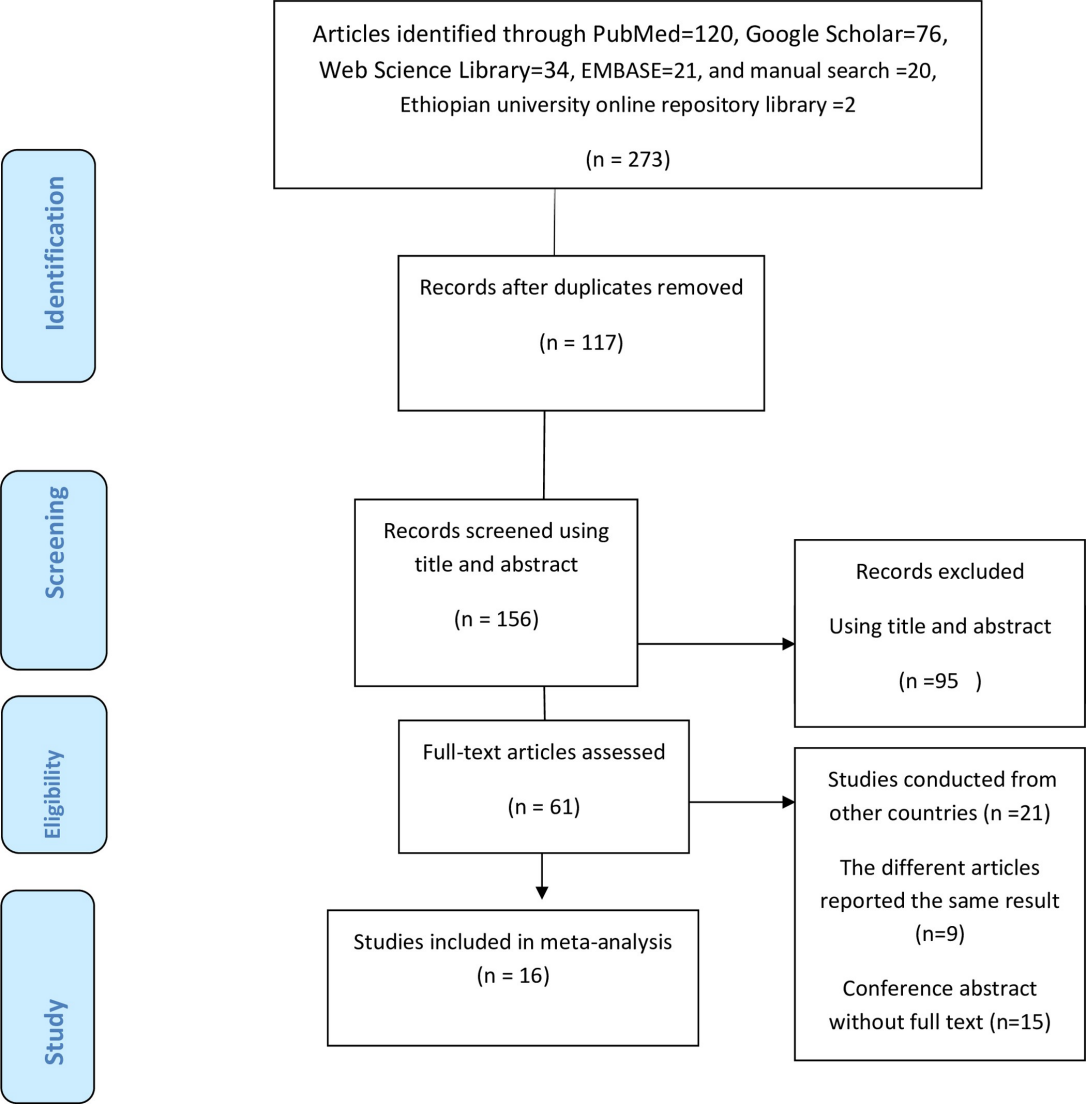
A Prisma diagrammatic presentation used to show the selection of studies. The inclusion criteria were variation of the title and abstracts, place of study (Ethiopia), presence of full abstract, and reporting different results. Studies were excluded if they criteria were duplicated source, unrelated research, case studies and qualitative studies.

There were 273 research articles retrieved using an electronic search. Of these articles, 117 were expelled for duplication and 95 studies were excluded after reviewing their titles and abstracts. At the qualification stage, 61 articles were completely gotten to and evaluated for the capability. Finally, 16 studies [48–63] with 12,475 participants were included in this systematic review and meta-analysis. All studies were cross-sectional, and reported the prevalence of knowledge, attitude, and practice of kangaroo mother care **(Fig 1).**

### Description of included studies

Out of 273 articles retrieved at first, sixteen articles met the eligibility criteria and were included in the final meta-analysis as reported by Fig 1. The author’s names, publication year, study design, sample size, study region, study setting, response rate, birth weight, kangaroo mother care type, gestational age, the prevalence of knowledge, attitude, and practice of kangaroo mother care listed in the below Table 1.

**Table 1:** Descriptions of the studies used in the systematic review and meta-analysis for the knowledge, attitude, and practice of kangaroo mother care among postnatal women in Ethiopia.

Author/year	Study region	Study Setting	Study Design	Sample Size	Response rate	Good knowledge	Good attitude	Good practice	GA	KMC Type	Weight (KG)	Study quality
Mose et.al/2021	SNNP	Hospital	Cross-sectional	382	100	35.5	50	35.3	Any age	SSC+BF	Any weight	Low risk
Gebre et.al/2018	Somali	Community	Cross-sectional	829	98.3	Not reported	Not reporter	23	Any age	SSC+BF	Any weight	Low Risk
Roba AA/2018	Harar & DireDawa	Hospital	Cross-sectional	349	100	69.91	63.33	54.51	Any age	SCC only	<2.5kg	Low Risk
Jamie, A.H /2020	Harar	Hospital	Cross-sectional	166	100	82.53	82.53	32.13	Any age	SSC only	<2.5kg	Low Risk
Alelign, Zewuditu(un-pub)	Addis Ababa	Hospital	Cross-sectional	249	100	69.1	54.22	43	Preterm	SSC+BF	<1.5kg	Low Risk
Bedaso et.al/2019	Amhara, Addis Ababa, Oromia	Hospital	Cross-sectional	384	100	Not reported	Not Reported	40.1	Any age	SSC	Any weight	Low Risk
Getinet et.al/2019	SNNP	Hospital	Cross-sectional	86	92	68.6	57	61.6	Preterm	SSC only	<2.5kg	Low Risk
Dawit, Aster (un-pub)	Addis Ababa	Hospital	Cross-sectional	297	100	Not reported	Not reporter	71	Preterm	SSC+BF	<1.5	Low Risk
Dabere et.al/2020	National	Community	Cross-sectional	7488	Not reported	Not reported	Not reported	24.3	Any age	SSC only	Any weight	Low Risk
Ebrahim yesuf et.al/2018	SNNP	Community	Cross-sectional	215	100	Not reported	Not reporter	41.9	Preterm	SSC only	<2.5kg	Low Risk
M.W,Ayele et.al/2021	Amhara	Community	Cross-sectional	190	97	Not reported	Not reported	46.8	Any age	SSC only	<2.5kg	Low Risk
Haftey Gebremedihn et,al (un-pub)	Tigray	Hospital	Cross-sectional	397	96.6	Not reported	Not reported	54.4	Any age	SSC only	<1.5kg	Low Risk
Lakew W. and B.Worku/2014	Addis Ababa	Hospital	Cross-sectional	110	Not reported	Not reported	Not reported	83	Preterm	SSC only	<1.5kg	Low Risk
Weldeargay et.al/2019	National	Hospital	Cross-sectional	768	Not reported	Not reported	Not reporter	46.4	Preterm	SSC only	<1.5kg	Low Risk
Demissie et.al/2018	Addis Ababa	Hospital	Cross-sectional	356	100	Not reported	Not reporter	47.2	Preterm	SSC+BF	<1.5kg	Low Risk
Emishaw et.al/	Tigray	Hospital	Cross-sectional	109	Not reported	Not reported	Not reporter	28.12	Preterm	SSC only	<2.5kg	Low Risk

Four studies were found in Addis Ababa [49, 53–55], three in Southern Nations Nationalities and Peoples Region [48, 50, 60], two at the national level [56, 58], one in Amhara [59], two in Tigray [57, 63], one in Somali [61], one in Harar and Dire Dawa [51], one in Harar [52], and one in Addis Ababa, Amhara, Oromia and Benishanguel Gumuize [62]. Of the sixteen cross-sectional studies, twelve were institutional-based, and four were community-based. The earliest in 2014 and the latest in 2021. The sample sizes ranged from 86 to 7488. The prevalence of knowledge, attitude, and practice of kangaroo mother care ranged were ranged from 35.5%-82.53%, 50%-82.53%, and 28%-83% respectively. The response rate ranged from 92 to 100 percent. Eight studies reported any gestational age (preterm, term, and post-term) infants, while the remaining only on preterm infants. All sixteen studies were assessed by using Joanna Briggs Institute (JBI) quality appraisal checklist. All of these studies had reported a low risk **(Table 1).**

### Level of knowledge, attitude, and practice towards kangaroo mother care

**Fig 2:**
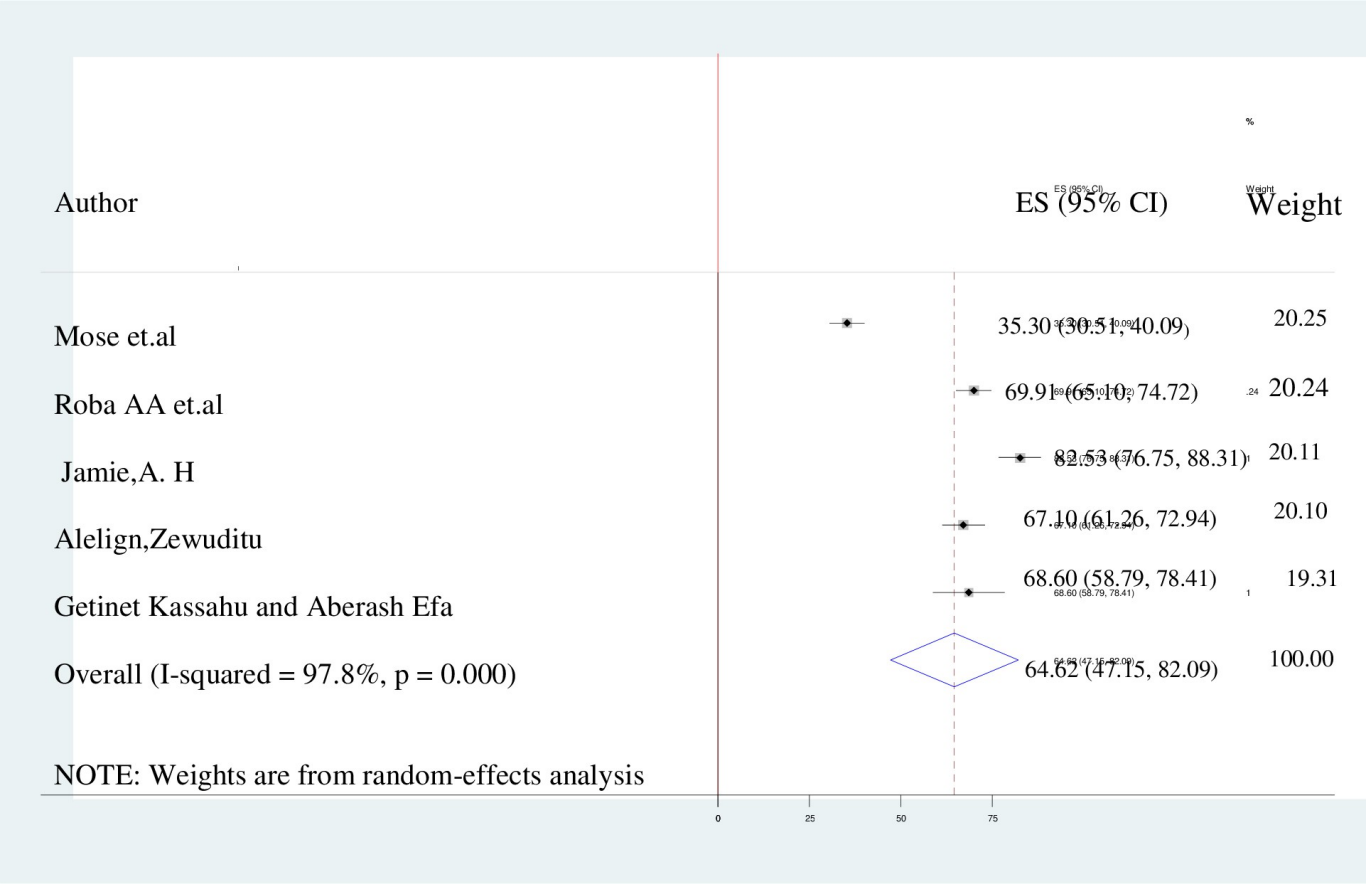
Forest plot of knowledge with the height of the diamond is the overall effect size (64.62% while the width is the confidence interval at 95% (47.15%–82.09%). The y-axis shows the standard error of each study while the x-axis the estimate of effect size of the each study. The vertical line denotes the no effect. The box represents the effect size of each study and the line across the box is confidence interval of each study.

**Fig 3:**
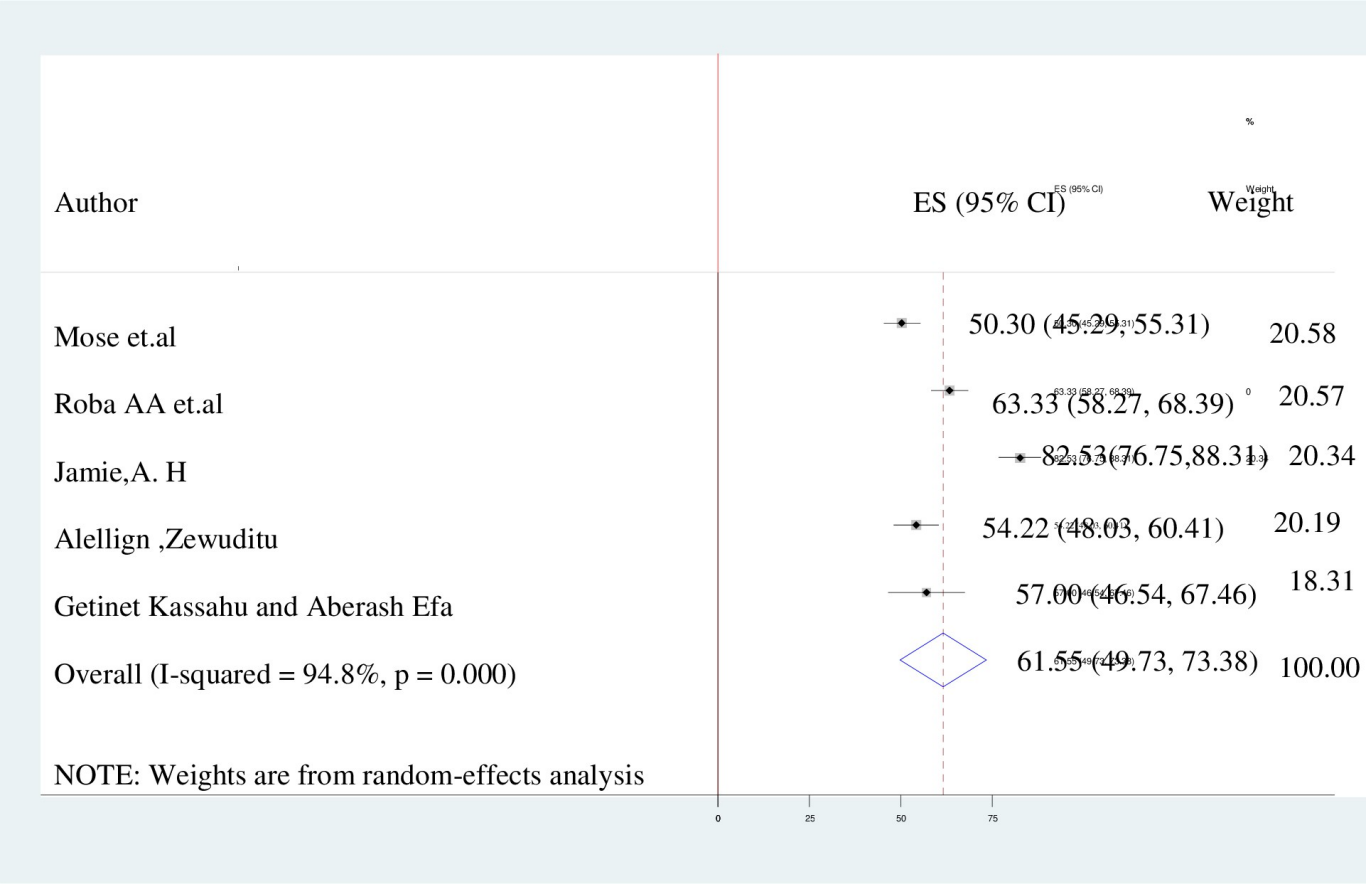
The forest plot of attitude with the diamond represents the summary point estimate (61.55%) and the horizontal extremity of the diamond is the confidence interval at 95% (49.73%–73.38). The standard error is plotted at the y-axis and the effect size plotted at x-axis. The squares represent the effect estimate of the individual studies and the horizontal lines indicate the confidence interval; the dimension of the square reflects the weight of each study.

**Fig 4:**
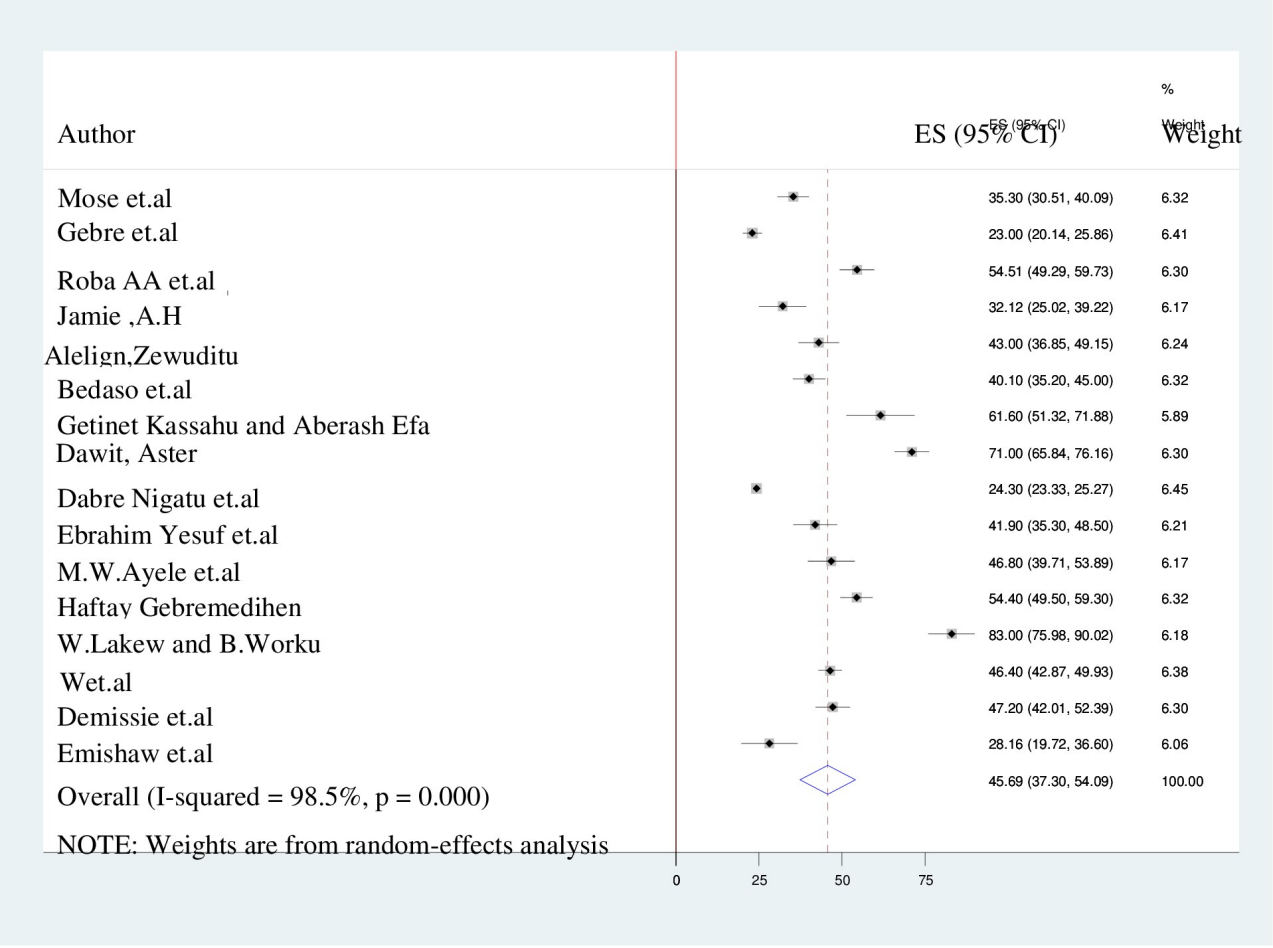
The forest plot of practice with the height of the diamond is the overall effect size (45.7%) while the width is the confidence interval at 95% (37.30%-54.09%). The y-axis shows the standard error of each study while the x-axis the estimate of effect size of the each study. The vertical line denotes the no effect. The square represents the effect size of each study and the line across the square is confidence interval of each study.

The pooled prevalence of the knowledge about, attitudes towards and practice of kangaroo mother care in Ethiopia is presented by the forest plots in **Figs 2–4**. A random-effect model showed that the pooled level of good knowledge was 64.62% (95% CI: 47.15%–82.09%; I^2^ = 97.8%). The overall estimated positive attitude towards kangaroo mother care was 61.55% (49.73%–73.38%; I^2^ = 94.8%), while the pooled estimate of poor practice of kangaroo mother care among postnatal women was 45.7% (95% CI: 37.30%–54.09%; I^2^ = 98.5%.

### Leave–one- out sensitivity analysis

A leave-one-out sensitivity analysis was carried out to detect the effect of each study on the overall prevalence of a good level of knowledge about, a positive attitude towards and a poor level of practice of kangaroo mother care among postnatal women by excluding one study at a time. The results showed that the excluded study leads to significant change in the overall prevalence of a good level of knowledge, positive attitude and poor practice. In the sensitivity analysis, both Jamie,AH. and Mose et al. showed an impact on the pooled level of good knowledge and positive attitude, while Lakew B, B. Worku and Gebre et al. showed an impact on the level of poor practice of kangaroo mother care (**Table 2**).

**Table 2:** A leave–out-one sensitivity analysis for knowledge, attitude, and practice of kangaroo mother care among postnatal women in Ethiopia.

Knowledge related articles		
Study omitted	Pooled estimate	95%CI
Mose et.al	72.22	64.92–79.53
Roba AA et.al.	63.31	40.30–86.32
Jamie, A.H	60.11	41.44–78.77
Zewuditu Alelign	64.02	41.59–86.45
Getinet et.al	63.67	43.23–84.12
Attitude related articles		
Study omitted	Pooled estimate	95%CI
Mose et.al	64.48	51.46–77.50
Roba AA et.al.	61.07	44.97–77.17
Jamie, A.H.	56.17	49.59–62.76
Alelign,zewuditu	63.39	48.82–77.96
Getinet et.al	62.58	48.82–76.33
Practice related articles		
Study omitted	Pooled estimate	95%CI
Mose.et.al	46.40	37.44–55.36
Roba AA et.al	45.10	36.52–53.68
M.W. Ayele et.al	45.62	36.90–54.35
Getinet et.al	44.70	36.12–53.28
Gebere et.al	47.26	37.83–56.69
Dawit, Aster	43.96	36.11–51.82
Bedaso et.al	46.08	37.17–54.98
Dabere et.al	47.16	38.94–55.38
Demissie et.al	45.60	36.83–54.36
Ebrahim Yesuf	45.95	37.17–54.73
Emishaw et.al	46.83	38.07–55.59
Jamie,A.H.	46.59	37.79–55.40
Zewditu Alelign	45.88	37.09–54.67
Tesfaye Geberemedihn	45.10	36.54–53.67
Weldearagay et.al	45.65	36.75–54.55
Lakew. W and B. Worku	43.22	35.37–51.07

### Subgroup analysis

The subgroup analysis based on kangaroo mother care component showed that a level of good knowledge was 74% in only skin-to-skin contact and 51.2% in skin-to-skin contact with exclusive breastfeeding. The level of positive attitude towards kangaroo mother care component was 67.98% in skin-to-skin contact only and 51.85% for skin-to-skin contact with exclusive breastfeeding. In this subgroup analysis, the level of poor practice was examined by study region, study setting, publication, component of kangaroo mother care, gestational age and birth weight. The pooled level of poor kangaroo mother care practice by region was 60.99% in Addis Ababa and 39.92% in another region (where a study conducted in single region/multiple regions). In the case of kangaroo mother care components, the prevalence of poor practice was 46.57% in only skin-to-skin contact practice and 43.84% in skin-to-skin contact with exclusive breastfeeding. Regarding gestational age, the level of poor practice was found to be 52.77% in preterm neonates and 38.68% at any gestational age. The level of poor practice relative to birth weight was 57.37% in birth weight below than 1.5 kg and 30.27% at any weight (**Table 3**).

**Table 3:** The overall estimated level of good knowledge, positive attitude, and poor practice towards kangaroo mother care in Ethiopia, 95%CI and heterogeneity estimate with a p-value for sub-group analysis.

Knowledge related articles			
Variable	Characteristics	Pooled level of good knowledge 95%(CI)	I^2^(p-value)
Kangaroo mother care type	SSC only	74% (64.744–83.236)	83.1%(0.003)
	SSC+BF	51.2%(19.991–82.318)	98.4% (0.000)
Attitude related articles			
Variable	Characteristics	Pooled level of positive Attitude 95%(CI)	I^2^(p-value)
Kangaroo mother care type	SSC only	67.98%(52.968–82.997)	93.5%(0.000)
	SSC+BF	51.85%(47.958–55.749)	0.0%(0.335)
Practice related articles			
Variables	Characteristics	Pooled level of poor practice 95%(CI)	I^2^(p-value)
Study setting	Community	33.067% (25.397–40.738)	95.4%(0.000)
	Institutional	49.697%(41.801–57.594)	95.9% (0.000)
Region	Addis Ababa	60.992%(43.326–78.658)	97.3%(0.000)
	SNNP	45.505%(32.660–58.349)	90.4%(0.000)
	Tigray	45.694%(37.297–54.092)	96.4%(0.002)
	Other	39.228%(26.286–52.170)	97%(0.002)
	Nationwide	35.282%(13.625–56.939)	99.3% (0.001)
Publication	Published	43.217% (34.943–51.492)	98.2%(0.000)
	Unpublished	56.200%(40.808–71.591)	95.9%(0.000)
Kangaroo mother care type	SSC only	46.572%(35.565–57.579)	98.6%(0.000)
	SSC+BF	43.844% (26.763–60.925)	98.6%(0.000)
Gestational age	Preterm	52.774%(41.867–63.681)	96.3%(0.000)
	Any age	38.675%(29.920–47.429)	97.9%(0.000)
Birth weight	<1.5kg	57.372%(46.223–68.522)	96.5%(0.000)
	1.5–2.5kg	44.080% (34.683–53.478)	90.3%(0.000)
	Any weight	30.274%(23.871–36.677)	94.8%(0.000)

### Publication bias

**Fig 5:**
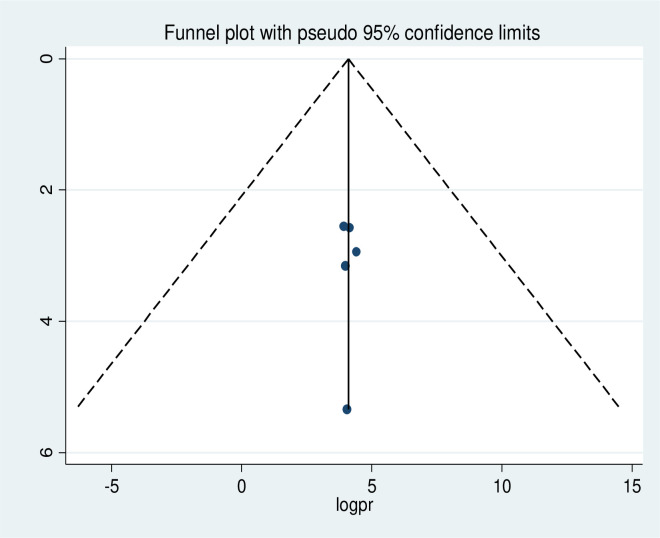
Funnel plot showing symmetrical distribution of studies indicating absence of publication bias. The Y-axis is the standard error and the X-axis is the study result or effect size. The dotted diagonal lie of the funnel is the 95% confidence interval and the vertical. The vertical line is the line of no-effect and dots are included studies reporting knowledge of kangaroo mother care.

**Fig 6:**
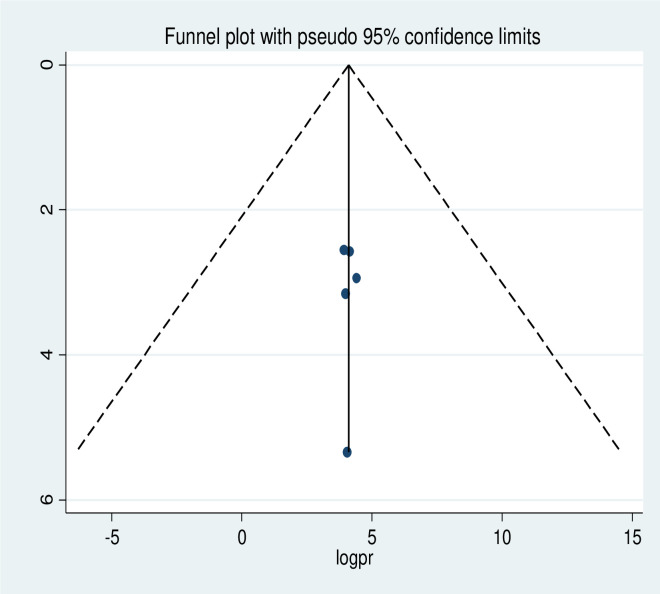
Funnel plot showing symmetrical distribution of studies indicating absence of publication bias. The Y-axis is the standard error and the X-axis is the study result or effect size. The dotted diagonal lie of the funnel is the 95% confidence interval and the vertical. The vertical line is the line of no-effect and dots are included studies reporting attitude of kangaroo mother care.

**Fig 7:**
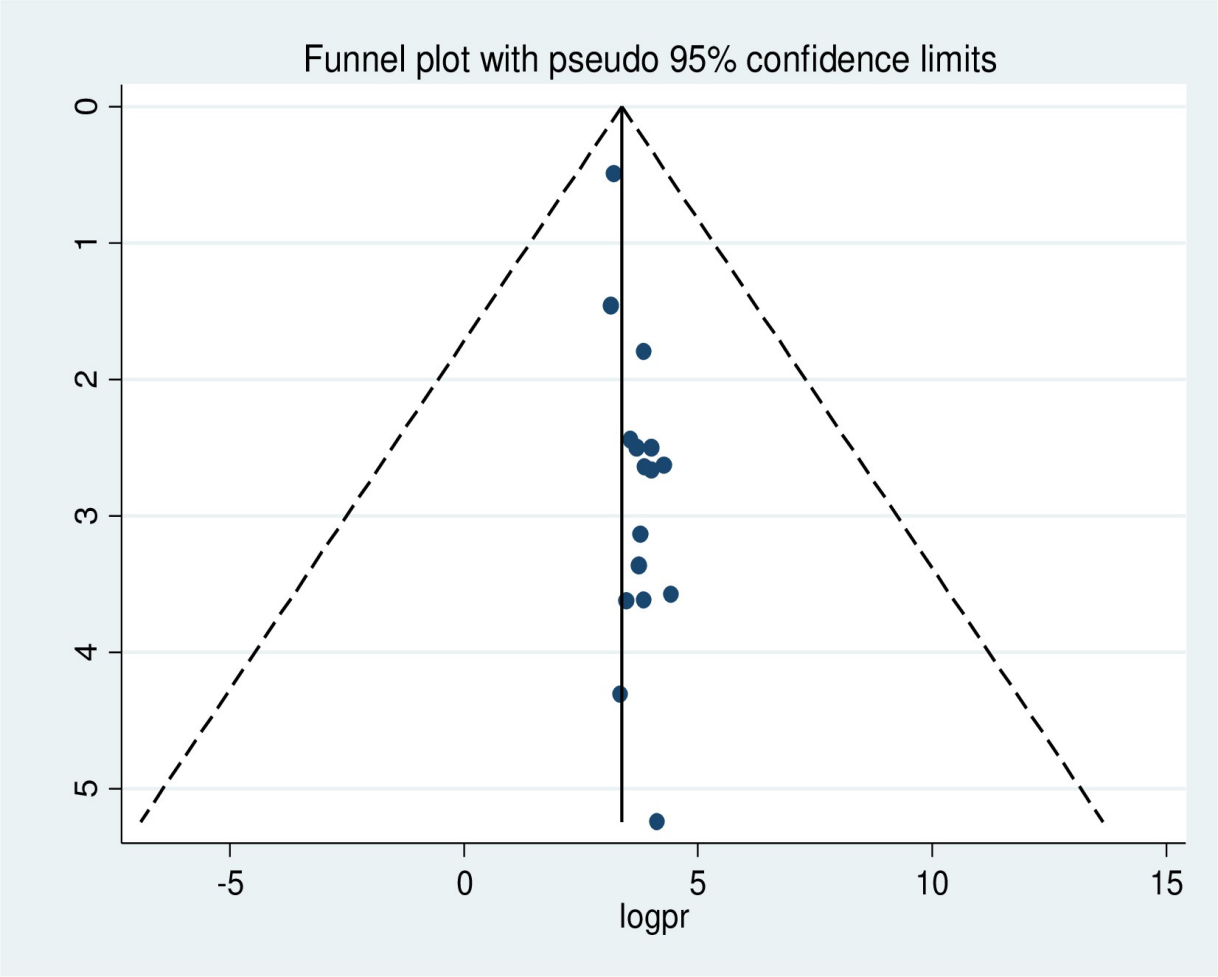
Funnel plot showing asymmetrical distribution of studies indicating the presence of publication bias. The Y-axis is the standard error and the X-axis is the study result or effect size. The dotted diagonal line of the funnel is the 95% confidence interval and the vertical. The vertical line is the line of no-effect and dots are included studies reporting attitude of kangaroo mother care.

**Fig 8:**
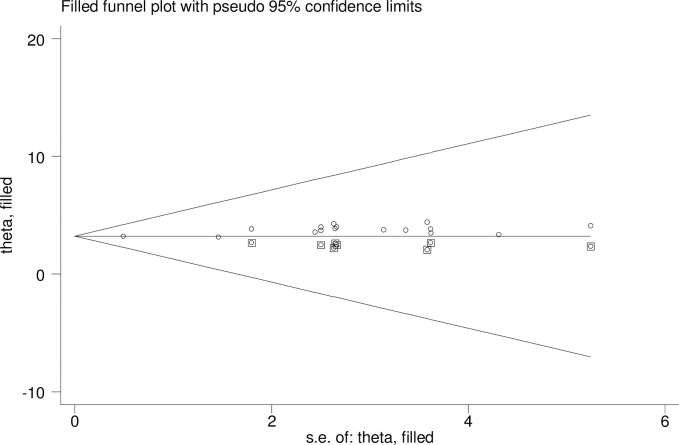
The funnel plot for trim-and-fill method was used to correct the result seven potential missing studies were required in the left side of the funnel plot to ensure symmetry. The enclosed circles represent the dummy studies and the free circles are genuine studies.

**Fig 9:**
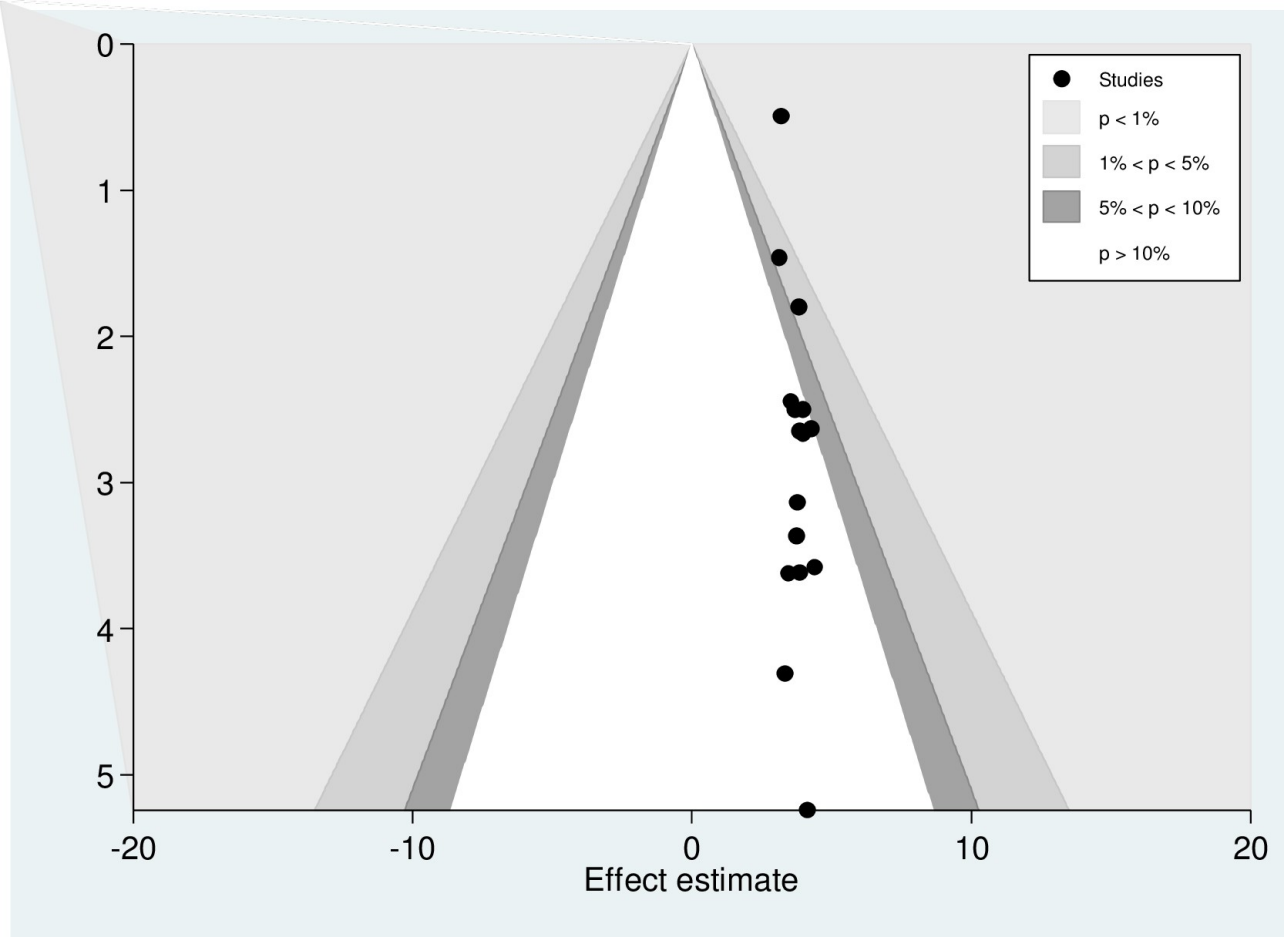
Counter-enhanced funnel plot suggestions of missing studies on the bottom left-hand-side of the plot. Since the majority of this area contains regions of high statistical significance (*P* < 0.01), this reduces the plausibility that publication bias is the underlying cause of this funnel asymmetry. Various shaded regions indicate statistical significance. In particular, the white shaded region in the middle corresponds to p-values greater than .10, the dark gray-shaded region corresponds to p-values between .10 and .05, the medium gray-shaded region corresponds to p-values between .05 and .01, and the region outside of the funnel corresponds to p-values below .01.

The presence of publication bias was checked using funnel plot visualisation and Egger’s and Begg’s regression tests (P<0.05). The Egger and Begg tests both revealed no statistical evidence of publication bias for a good level of knowledge (P = 0.577 and P = 0.240, respectively; **Fig 5**). There was also no statistical evidence of publication bias for a positive attitude in terms of the Egger (P = 0.928) and Begg (p = 0.624) tests (**Fig 6**). The results of the Begg (P = 0.000) and Egger (P = 0.000) tests show the presence of publication bias for the level of poor practice of kangaroo mother care. Additionally, an asymmetric distribution was visualised on the funnel plot (**Fig 7)**. Subsequently, trim-and-fill analysis was performed and indicated the presence of seven unpublished studies (**Fig 8**). A counter-enhanced funnel plot was also calculated, and the missing studies in the areas of higher statically significance suggested that the cause of asymmetry was due to factors other than publication bias, such as the study variables (**Fig 9**).

## Discussion

Kangaroo mother care was first introduced in Ethiopia in 1996 at the Black Lion Hospital in Addis Ababa. Since then, kangaroo mother care services have expanded to other hospitals and health facilities at all levels. Nowadays, they have been issued in a series of policy documents by the Federal Ministry of Health, the New born and Child Survival Strategy 2015–2020, the Health Sector Transformation Plan, and the National Healthcare Quality Strategy. Despite a governmental emphasis on reducing neonatal mortality via evidence-based strategies such as kangaroo mother care, Ethiopia is one of the sub-Saharan African countries with the most neonatal mortalities.

Due to the scant availability of literature, we conducted this systematic review and meta-analysis to better understand the knowledge, attitudes and practices among Ethiopian women. We included all available studies using a variety of electronic search engines and were also able to undertake a sub-group analysis assessing the proportion of knowledge, attitudes and practices by study setting, study region, gestational age, component of kangaroo mother care, birth weight and publications.

This study’s findings reveal that the pooled prevalence of good kangaroo mother care knowledge was 64.62%. This is lower than the results of a study conducted in the USA (75%) among nurses working in intensive care units [66]. This inconsistency can be attributable to existing socio-economic, healthcare system infrastructure, study population variation, and methodological differences (including study design) across the studies. The sub-group analysis, based on components of kangaroo mother care, showed that participants were more knowledgeable concerning skin-to-skin contact only compared to both skin-to-skin contact and exclusive breastfeeding. This finding is not surprising because exclusive breastfeeding has received much attention lately.

More than half (61.5%) % of women who gave birth in Ethiopia had a positive attitude towards kangaroo mother care. This finding is consistent with a US result, which showed that over 60% of respondents had a positive perception of kangaroo mother care [66]. The sub-group analysis also revealed that participants had a positive attitude towards skin-to-skin contact only over both skin-to-skin contact and exclusive breastfeeding, which might be due to the lack of long-term exposure to exclusive breastfeeding.

The pooled level of poor kangaroo mother care practice was 45.7%. This was relatively lower than the findings of studies conducted in New Zealand (69%) and Singapore (92%) [67, 68]. One conceivable clarification for the disparity is the methodological differences, such as study design and socio-cultural difference, between the countries, which, in turn, affect the practice of kangaroo mother care. Furthermore, Ethiopia is a third-world country with a poor economic status and poor maternal and paediatric health coverage, along with low levels of skilled deliveries. The sub-group analysis showed that poor practices were observed in the community-based study, skin-to-skin contact and exclusive breastfeeding (at any weight or gestational age); in contrast, a high level of practice was reported in Addis Ababa. This indicates the significance of kangaroo mother care for very-low-birth-weight, low-birth-weight and preterm neonates, as well as the need to enhance the procedure at the community level. Regarding region, Addis Ababa had a greater share of kangaroo mother care practice, which might be due to the adaptation of the procedure by both mothers and healthcare providers for a long time since its introduction at the Black Lion Hospital (one of Ethiopia’s earliest specialised hospitals), located in Addis Ababa, Ethiopia.

In this study, we utilised a random-effect model to manage a significant variation that resulted in between-study heterogeneity. We assessed leave-one-out sensitivity and the results show that every study had a significant impact on the pooled good level of knowledge, positive attitude, and poor level of kangaroo mother care practice. We also assessed the possible variability source via sub-group analysis using the study settings, regions, publications, gestational ages, birth weights and components of kangaroo mother care. The high heterogeneity might be due to differences in the sample populations, paper qualities, or socio-cultural, ethnic and regional differences.

## Conclusion

In conclusion, this systematic review and meta-analysis reported that there was a significant gap in knowledge, attitudes, and practices of kangaroo mother care among Ethiopian women. Besides, the pooled prevalence of knowledge, attitudes, and practices differed based on the study settings, regions, publications, gestational ages, birth weights and components of kangaroo mother care. The information generated from these findings should be used for the provision of accurate and up-to-date training and education of kangaroo mother care. Accordingly, it is better to have periodic kangaroo mother care training for postnatal women and, subsequently, expand service dimensions across the community level.

### Strength and limitation of the study

The strength of the study including the use of a comprehensive electronic search strategy through the variety of datasets to determine the overall level of knowledge, attitude, and practice of kangaroo mother care, the use of JBI-MAStARI appraisal, and the accessing of grey literature’s. This study also had some limitations. These were the absence of a standard definition of good knowledge, positive attitude, and poor practice of kangaroo mother care to operationalize by the research team and might be researched bias on a cut of point. The absence of a similar previous study makes it is very difficult to compare the findings of this study. The study had included the whole regions of the country, but Afar and Gambela.

## Supporting information

S1 File Prisma checklist. (PDF)

S2 File Methodological quality assessment of included studies using Joanna Brigg’s Institute quality appraisal criteria scale (JBI). The eight item questions assessing inclusion criteria, study setting and participant, exposure measurement, objectives, confounder, statically analysis, outcome measurement and dealing confounder were used.
(PDF)

S3 File Risk of bias assessment for the included studies. The ten item questions of which four items assess external and six items assess internal validity were used.
(PDF)

## References

[1] WHO and UNICEF Joint Statement. (2009). Home visits for a newborn child: a strategy to improve survival 24809117

[2] Oza S, Cousens S, Lawn J. Estimation of daily risk of neonatal death, including the day of birth, in 186 countries in 2013: A vital-registration and modeling-based study. Lancet Glob Health. 2014;2:e635–44. doi: 10.1016/S2214-109X(14)70309-2 25442688

[3] Lawn J, Blencowe H, Oza S, You D, Lee A, Waiswa P, et al. Progress, priorities, and potential beyond survival. Lancet. 2014;384:189–205. 10.1016/S0140-6736(14)60496-7 24853593

[4] UN Inter-agency Group for Child Mortality Estimation. Levels & trends in child mortality: report 2019. New York: UNICEF; 2019. https://www.unicef.org/media/60561/file/UN-IGME-child-mortality-report-2019.pdf. Accessed 14 Oct 2019

[5] Beck S, Wojdyla D, Say L, Betran AP, Merialdi M, Requejo JH, et al. (2010). The worldwide incidence of preterm birth: a systematic review of maternal mortality and morbidity. Bull World Health Organ, 88(1):31–38 doi: 10.2471/BLT.08.062554 20428351 PMC2802437

[6] Gomez HM, Sanabria ER, Marquette CM. The Mother Kangaroo Programme. International Child Health, (1992). 3(1):55–67.

[7] Muchie et al. Epidemiology of preterm birth in Ethiopia: systematic review and meta-analysis: BMC Pregnancy and Childbirth (2020) 20:574 doi: 10.1186/s12884-020-03271-6 32993555 PMC7526155

[8] Endalamaw et al. Low birth weight and its associated factors in Ethiopia: a systematic review and meta-analysis: Italian Journal of Pediatrics (2018) 44:141 doi: 10.1186/s13052-018-0586-6 30477557 PMC6258299

[9] Lee A. C. C., Blencowe H., and Lawn J. E., “Small babies, big numbers: global estimates of preterm birth,” The Lancet Global Health, vol. 7, no. 1, pp. e2–e3, 2019 doi: 10.1016/S2214-109X(18)30484-4 30389450

[10] World Health Organization. Kangaroo Mother Care: a Practical Guide. WHO, Geneva, Switzerland 2003.

[11] World Health Organization. Guideline: protecting, promoting, and supporting breastfeeding in facilities providing maternity and newborn services. World Health Organization; 2017. Available from: https://apps.who.int/iris/bitstream/handle/10665/259386/9789241550086-eng.pdf. Accessed June 8, 2021. 29565522

[12] Baley J. Skin-to-skin care for the term and preterm infants in the neonatal ICU. *Pediatrics*. 2015;136(3):596–599. doi: 10.1542/peds.2015-2335 26324876

[13] Feldman-Winter L, Goldsmith JP, Moon RY, et al. Safe sleep and skin-to-skin care in the neonatal period for healthy term newborns. *Pediatrics*. 2016. doi: 10.1542/peds.2016-1889 27550975

[14] Abdulghani N, Edvardsson K, Amir LH, van Wouwe JP. Worldwide prevalence of mother-infant skin-to-skin contact after vaginal birth: a systematic review. *PLoS One*. 2018;13(10):e0205696. doi: 10.1371/journal.pone.0205696 30379859 PMC6209188

[15] Broughton E, Gomez I, N S, Vindell C. The cost-savings of implementing kangaroo mother care in Nicaragua 2013. 24233110

[16] Bigelow A, Power M, MacLellan-Peters J, Alex M, McDonald C. Effect of mother/infant skin-to-skin contact on postpartum depressive symptoms and maternal physiological stress 2012. J Obstet Gynecol Neonatal Nurs 41: 369–382 doi: 10.1111/j.1552-6909.2012.01350.x 22537390

[17] Conde-Agudelo AD-RJ. Kangaroo mother care to reduce mortality in low birth weight infants. Cochrane Database Syst Review. 2014;22(4) 10.1002/14651858.CD002771.pub3 24752403

[18] A-M B. In: WRHL G, editor. Kangaroo mother care to reduce morbidity and mortality in low-birth-weight infants; 2011 10.1111/ijn.12541 28513087

[19] Vesel L, Bergh A-M, Kerber KJ, et al. Kangaroo mother care: a multi-country analysis of health system bottlenecks and potential solutions. BMC Pregnancy Childbirth. 2015;15 doi: 10.1186/s12884-015-0437-z 26391115 PMC4577801

[20] Bhutta ZA, Das JK, Bahl R, Lawn JE, Salam RA, Paul VK, et al. Can available interventions end preventable deaths in mothers, newborn babies, and stillbirths, and at what cost? Lancet. 2014;384:347–70 doi: 10.1016/S0140-6736(14)60792-3 24853604

[21] Lassi ZS, Middleton PF, Crowther C, Bhutta ZA. Interventions to improve neonatal health and later survival: an overview of systematic reviews. EBio Medicine. 2015; 2 (8) doi: 10.1016/j.ebiom.2015.05.023 26425706 PMC4563123

[22] World Health Organization. WHO recommendations on interventions to improve preterm birth outcomes. Geneva: World Health Organization; 2015 26447264

[23] Boundy E, Dastjerdi R, Spiegelman D, Wafaie W. Kangaroo Mother Care, and neonatal outcomes: a meta-analysis. Pediatrics. 2016. 10.1542/peds.2015-2238 PMC4702019 26702029

[24] Karimi F.Z. et al. The effect of mother-infant skin to skin contact on success and duration of first breastfeeding: A systematic review and meta-analysis: Taiwanese Journal of Obstetrics & Gynecology 58 (2019) doi: 10.1016/j.tjog.2018.11.002 30638460

[25] Mekonnen et al. The effects of kangaroo mother care on the time to breastfeeding initiation among preterm and LBW infants: a meta-analysis of published studies: International Breastfeeding Journal (2019) 14:12 doi: 10.1186/s13006-019-0206-0 30820239 PMC6379962

[26] Karimi et al. The effect of mother-infant skin-to-skin contact immediately after birth on exclusive breastfeeding: a systematic review and meta-analysis: J Turk Ger Gynecol Assoc 2020; 21: 46–56 doi: 10.4274/jtgga.galenos.2019.2018.0138 30905140 PMC7075405

[27] Ghojazadeh M, Hajebrahimi S, Pournaghi-Azar F, Mohseni M, Derakhshani N, Azami Aghdash S. Effect of kangaroo mother care on successful breastfeeding: a systematic review and meta-analysis of randomized controlled trials. RevRecent Clinical Trials. 2019; 14:31 _40. 10.2174/1574887113666180924165844 30251612

[28] Vaivada T, Gaffey MF, Bhutta ZA. Promoting early child development with interventions in health and nutrition: a systematic review.Pediatrics.2017;140:e20164308. doi: 10.1542/peds.2016-4308 28771408

[29] Sharma D, Farahbakhsh N, Sharma S, Sharma P, Sharma A.Role of kangaroo mother care in growth and breastfeeding rates in very low birth weight neonates: a systematic review. J Maternal-Fetal Neonatal Med.2019;32:129 _42. doi: 10.1080/14767058.2017.1304535 28274153

[30] Cong et al. Skin-to-skin contact to improve premature mothers’ anxiety and stress state: A meta-analysis: maternal and child nutrition, Willey 2021 reviewed article doi: 10.1111/mcn.13245 PMC8476413 34258864

[31] Akbari E. et.al Kangaroo mother care and infant bio-psychosocial outcomes in the first year: A meta-analysis: Elsevier; a journal of Early Human Development 122 (2018) 22 10.1016/j.earlhumdev.2018.05.004 29843051

[32] Boundy EO, Dastjerdi R, Spiegelman D, et al. Kangaroo Mother Care, and Neonatal Outcomes: A Meta-analysis. *Pediatrics*. 2016;137(1):e20152238 10.1542/peds.2015-2238 PMC4702019 26702029

[33] Jafari M, Farajzadeh F, Asgharlu Z, Derakhshani N, Asl YP. Effect of Kangaroo Mother Care on hospital management indicators: A systematic review and meta-analysis of randomized controlled trials. J Edu Health Promot 2019;8:96. doi: 10.4103/jehp.jehp_310_18 31143813 PMC6532364

[34] Macaraya JRG, Padua AP and Dans LF. Skin to skin contact as analgesia for acute procedural pain in neonates:meta-analysis: Philipins Journal of Pediatrics vol.64.no.1 2016

[35] Shrivastava S.R., Shrivastava P.S., Ramasamy J., 2013. Utility of kangaroo mother care in preterm and low birth weight infants. S. Afr. Fam. Pract. 55 (4), 340–344. doi: 10.1080/20786204.2013.10874373, 2013.

[36] Vohra A.S., Shah B.H., Mehariya K.M., 2017. Effect of kangaroo mother care on feeding, morbidity, and neurodevelopment of low birth weight neonates. Int. J. Contemp. Med. Res. 4, 1029–1032.

[37] Sharma D, Murki S, and Oleti TP. Study comparing. Kangaroo Ward Care" with" Intermediate Intensive Care "for improving the growth outcome and cost-effectiveness: randomized control trial. J Matern Fetal Neonatal Med.2018;31:2986 _93 doi: 10.1080/14767058.2017.1359832 28738711

[38] Acharya N, Singh RR, Bhatta NK, Poulde lP. Randomized control trial of kangaroo mother care in low birth weight babies at a tertiary level hospital. J Nepal Paediatr Soc. 2014;34:18 _23

[39] Lumbanraja SN. Influence of maternal factors on the successful outcome of kangaroo mother care in low birth-weight infants: a randomized controlled trial. JNeonatal PerinatalMed.2016; doi: 10.3233/NPM-161628 28009335

[40] Mwendwa AC, Musoke RN, Wamalwa DC.Impactofpartialkan-karoo mother care on growth rates and duration of hospital stay of low birth weight infants at the Kenyatta National Hospital, Nairobi. EastAfrMedJ.2012;89:53 _8. 26845812

[41] Kumbhojkar S, Mokase Y, Sarawade S. Kangaroo mother care: an alternative to the conventional method of care for low birth weight babies. IntJHealth SciRes.2016;6:36 _42

[42] Ministry of Health. (2015). Ethiopian National Health Care Quality Strategy 2016–2020. www.medbox.org/et-policies-others/ethiopian-national-health-care-quality-strategy-2016-2020/preview

[43] Ministry of Health. (2015). Health Sector Transformation Plan (HSTP). www.Researchgate.net/file.PostFileLoader.html?id=56efcc94cbd5c2c1930fae21&assetKey=AS%3A342023027609601%401458556052424

[44] Ministry of Health. (2015). Newborn and Child Survival Strategy Document Brief Summary 2015/16-2019/20. www.unicef.org/ethiopia/Child_Survival_Strategy.pdf

[45] Hug L, Alexander M, You D, Alkema L. National, regional, and global levels and trends in neonatal mortality between 1990 and 2017, with scenario-based projections to 2030: a systematic analysis. *Lancet Glob Health*. 2019;7(6) 10.1016/S2214-109X(19)30163-9 PMC6527519 31097275

[46] Ethiopian Public Health Institute (EPHI)[Ethiopia] and ICF. Ethiopia mini demographic and health survey 2019: key indicators.

[47] Penfold S, Hill Z, Mrisho M, et al. A large cross-sectional community-based study of newborn care practices in Southern Tanzania. *PLoS One*.2010;5(12) doi: 10.1371/journal.pone.0015593 21203574 PMC3006340

[48] Mose et al. Skin-to-Skin Care Practice and Its Associated Factors among Postpartum Mothers in Gurage Zone, Southern Ethiopia: A Cross-Sectional Study: Pediatric Health, Medicine and Therapeutics 2021 doi: 10.2147/PHMT.S306411 34163284 PMC8216067

[49] Alelign Zewditu. Assessment of knowledge, attitude, practice and associated factors towards kangaroo mother care among postnatal mothers paired with their babies at Addis Abeba Public hospitals, 2019: a cross-sectional study: Addis Ababa university institutional repository http://etd.aau.edu.et/handle/123456789/21465/Zewditu%20alelign.pdf?sequence=1&isAllowed=y

[50] Getinet et.al. Knowledge, Attitude, Practice and Associated Factors of Kangaroo Mother Care for Neonatal Survival Among Care Takers of Preterm and Low Birth Weight Infants in Health Care Settings, Hawassa, Southern Ethiopia2019: Journal of Health, Medicine and Nursing

[51] Roba et al. Knowledge, Attitude and Practice of Kangaroo Mother Care by Postnatal Mothers who Gave Birth to Preterm and Low Birth Weight Babies in Public Hospitals, Eastern Ethiopia: J Neonatal Biol 2017 doi: 10.4172/2167-0897.1000257 30294505 PMC6169804

[52] Jamie A. H. Knowledge, attitude and practice of kangaroo mother care among postnatal mothers in a tertiary care center of Harari region, Harar, Ethiopia. *Public Health of Indonesia*, (2020). 6(4),116–122.

[53] Dawit Aster. Dawit Aster (2015) Assessment of Exclusive Breast Feeding Practice and Factors Affecting it among Mothers who Provided Kangaroo Mother Care to their Preterm Infants in Black Lion Hospital, Addis Ababa, Ethiopia. Masters thesis, Addis Ababa University.Available at http://etd.aau.edu.et/bitstream/handle/123456789/7614/Aster%20Dawit%20final%20thesis.pdf?sequence=1

[54] Demissie et al. Neonatal hypothermia and associated factors among neonates admitted to neonatal intensive care unit of public hospitals in Addis Ababa, Ethiopia: BMC Pediatrics (2018) 18:263 doi: 10.1186/s12887-018-1238-0 30077179 PMC6090740

[55] Lakew W., Worku B. Follow-Up Profile and Outcome of Preterm Managed with Kangaroo Mother Care: Open Journal of Pediatrics, 2014, 4, 143–147

[56] Dabre Nigatu et.al. Maternal Health Service Uptake Is Associated with a Higher Skin-to-Skin Care Practice in Ethiopia: Result from a National Survey: BioMed Research International Volume 2020, Article ID 8841349, 10 pages 10.1155/2020/8841349 PMC7768584 33415162

[57] Gebremedhin Haftay, Hagos Degefa Hidru, Berihu Gidey, Medhin Mehari, Meresa Berwo and Muzayene Tilahun. Kangaroo_mother_care_practice_and_its_associated_factors_among_postnatal_mothers_who_gave_birth_in_public_general_hospitals_of_Eastern_and_Central_zone_Tigray_North_Ethiopia: a [preprint] [cited August 17th, 2020] available from: https://doi.org10.21203/rs.3.rs-57242/v1

[58] Weldearegay HG, Medhanyie AA, Abrha MW, Tadesse L, Tekle E, Yakob B, et al. 2019. Quality of Kangaroo Mother Care services in Ethiopia: Implications for policy and practice. PLoS ONE 14(11): doi: 10.1371/journal.pone.0225258 31756225 PMC6874352

[59] Ayele M.W. et al. Proportion of mothers practicing kangaroo mother care at home and factors influencing the practices following hospital discharge, South Wollo, Ethiopia: Journal of Neonatal Nursing 27 (2021) 118–122

[60] Ebrahim Yusuf et.al. Utilization of Kangaroo Mother Care (KMC) and Influencing Factors Among Mothers and Care Takers of Preterm/Low Birth Weight Babies in Yirgalem Town, Southern, Ethiopia: Diversity and Equality in Health and Care (2018) 15(2): 87–92

[61] Gebre et.al. Newborn-Care Practices and Health-Seeking Behavior in Rural Eastern Ethiopia: A Community-Based Study: Journal of Tropical Pediatrics, 2018, 64, 90–96 doi: 10.1093/tropej/fmx031 28549190

[62] Bedaso et al. Assessment of skin‑to‑skin contact during the postpartum stay and its determinant factors among mothers at public health institutions in Ethiopia: BMC Res Notes (2019) 12:136 doi: 10.1186/s13104-019-4176-5 30871623 PMC6417157

[63] Emishaw et.al. Mothers Practice and Associated Factors on Kangaroo Mother Care, in Mekelle City Hospitals Tigray, North Ethiopia: A Cross-Sectional Survey: Research & Reviews: A Journal of Immunology

[64] Moher D, Liberati A, Tetzlaff J, Altman DG, The PRISMA Group (2009). The Preferred Reporting Items for Systematic Reviews and Meta-Analyses: The PRISMA Statement. PLoS Med 6(7): e1000097. doi: 10.1371/journal.pmed.1000097 19621072 PMC2707599

[65] Hoy D, Brooks P, Woolf A, Blyth F, March L, Bain C, et al. Assessing risk of bias in prevalence studies: modification of an existing tool and evidence of interrater agreement. J Clin Epidemiol. 2012;65(9):934–9. doi: 10.1016/j.jclinepi.2011.11.014 22742910

[66] Engler Arthur J., Ludington-Hoe Susan M., Cusson Regina M., Adams Rene, Bahnsen Millie, Brumbaugh Eileen, et al. National Survey of Practice, Knowledge, Barriers, and Perceptions: The American Journal of Maternal/Child Nursing · May 2002 10.1097/00005721-200205000-00004 12015442

[67] Kalmakoff S, Gray A, Baddock S. Predictors of supplementation for breastfed babies in a Baby-Friendly hospital. Women and Birth. 2017. doi: 10.1016/j.wombi.2017.08.131 28888864

[68] Lau Y, Tha PH, Ho-Lim SST, Wong LY, Lim PI, Citra Nurfarah BZM, et al. An analysis of the effects of intrapartum factors, neonatal characteristics, and skin-to-skin contact on early breastfeeding initiation. Maternal & Child Nutrition. 2017:e12492. 10.1111/mcn.12492 PMC6866048 28799193

