# Supplementary material for: Knowledge, attitude and practice towards kangaroo mother care among postnatal women in Ethiopia: Systematic review and meta-analysis
Source: PLoS One. 2022 May 6;17(5):e0265411. doi: 10.1371/journal.pone.0265411 (PMC9075620; doi:10.1371/journal.pone.0265411)
Supplement: S2 File — The eight item questions assessing inclusion criteria, study setting and participant, exposure measurement, objectives, confounder, statically analysis, outcome measurement and dealing confounder were used. (PDF) [file pone.0265411.s002.pdf]

Table 1: Methodological quality assessment of included studies using Joanna Brigg's Institute quality appraisal criteria scale (JBI)

For cross-sectional study

Item	Getinet et.al [51]	Mose et.al [49]	Roba AA. et.al [52]	Ebrahim et.al [61]	Enishaw et.al [64]	Dawit Aster [54]	Gebere et.al [62]	Bedaso et.al [63]	Dabere et.al [57]	Jamie A.H [53]	Alelign, Zewditu [50]	Geberemedihn et.al [58]	Woldearagay Et.al [59]	Lakew.w & B.Worku [56]	Demisie et.al [55]	M.W. Ayele et.al [60]
Clearly defined inclusion criteria	Yes	Yes	Yes	Yes	Yes	Yes	Yes	Yes	Yes	No	Yes	No	No	No	Yes	Yes
Describing the study settings and participants	Yes	Yes	Yes	Yes	Yes	Yes	Yes	Yes	Yes	Yes	Yes	Yes	Yes	Yes	Yes	Yes
Valid and reliable exposure measurement	No	Yes	Yes	Yes	Yes	No	No	No	No	Yes	No	Yes	Yes	Yes	No	Yes
Objective and standard criteria for measurement	Yes	Yes	Yes	Yes	Yes	Yes	Yes	Yes	Yes	Yes	Yes	Yes	Yes	Yes	Yes	Yes
Identified confounder	No	No	No	Yes	No	No	No	Yes	Yes	No	No	No	Yes	No	Yes	No
Strategies to deal with confounder	No	No	No	Yes	No	No	No	Yes	Yes	No	No	No	Yes	No	Yes	No
Valid and reliable outcome measurement	Yes	Yes	No	No	No	Yes	Yes	No	Yes	Yes	Yes	Yes	Yes	Yes	Yes	No
Appropriate statistical analysis	Yes	Yes	Yes	Yes	Yes	Yes	Yes	Yes	Yes	Yes	Yes	Yes	Yes	Yes	Yes	Yes
**Percentage of yes (%)**	5/8=62.5	6/8=75	5/8=62.5	7/8=87.5	6/8=75	5/8=62.5	5/8=62.5	6/8=75	7/8=87.5	5/8=62.5	5/8=62.5	5/8=62.5	7/8=87.5	5/8=62.5	7/8=87.5	5/8=62.5
