# Supplementary material for: Knowledge, attitude and practice towards kangaroo mother care among postnatal women in Ethiopia: Systematic review and meta-analysis
Source: PLoS One. 2022 May 6;17(5):e0265411. doi: 10.1371/journal.pone.0265411 (PMC9075620; doi:10.1371/journal.pone.0265411)
Supplement: S3 File — The ten item questions of which four items assess external and six items assess internal validity were used. (PDF) [file pone.0265411.s003.pdf]

Table 2.risk of bias assessment for the included studies

Item	Getinet et.al [51]	Roba AA et.al [52]	M.W. Ayele et.al [60]	Mose et.al [49]	Gebere et.al [62]	Dawit Aster [54]	Bedaso et.al [63]	Dabere et.al [57]	Demise et.al [55]	Ebrahim et.al [61]	Enishaw et.al [64]	Jamie. A.H [53]	Alelign ,Zewud itu [50]	Geberemedih et.al [58]	Woldearaga y et.al [59]	Lakew.w & B. Worku [56]
**External validity**																
Representativeness of the target population	Yes	Yes	Yes	Yes	Yes	Yes	Yes	Yes	Yes	No	Yes	Yes	Yes	Yes	Yes	Yes
Representativeness of the sampling frame	Yes	Yes	No	Yes	Yes	No	Yes	Yes	Yes	No	Yes	No	Yes	Yes	Yes	Yes
Radom sampling or census	Yes	Yes	No	No	No	No	Yes	No	Yes	Yes	Yes	No	Yes	Yes	Yes	Yes
Minimal response bias	Yes	Yes	Yes	Yes	Yes	yes	Yes	Yes	Yes	Yes	Yes	Yes	Yes	Yes	Yes	Yes
**Internal validity**																
Data were collected directly	Yes	Yes	Yes	Yes	No	Yes	Yes	No	No	Yes	Yes	Yes	Yes	Yes	No	Yes
Acceptable case definition used in the study	No	Yes	Yes	Yes	No	Yes	No	Yes	Yes	Yses	No	Yes	Yes	Yes	Yes	No
Valid and reliable measurement	Yes	No	Yes	Yes	Yes	Yes	No	Yes	Yes	Yes	Yes	Yes	Yes	Yes	Yes	No
The same mode of data collection for all study subject	No	No	Yes	Yes	Yes	Yes	Yes	Yes	No	Yes	Yes	Yes	Yes	No	yes	Yes
Appropriate length of prevalence period for parameter of interest	Yes	Yes	Yes	Yes	Yes	Yes	Yes	Yes	Yes	Yes	Yes	Yes	Yes	Yes	Yes	Yes
Appropriate numerators and denominators of interest	Yes	Yes	Yes	Yes	Yes	Yes	Yes	Yes	Yes	Yes	Yes	Yes	No	Yes	Yes	Yes
No of yes	8	8	8	9	8	8	8	9	8	8	9	8	9	9	9	8
**Summary of risk of bias**	Low-risk	Low-risk	Low-risk	Low-risk	Low-risk	Low-risk	Low-risk	Low-risk	Low-risk	Low-risk	Low-risk	Low-risk	Low-risk	Low-risk	Low-risk	Low-risk
