# Supplementary material for: Knowledge, attitude and practice towards kangaroo mother care among postnatal women in Ethiopia: Systematic review and meta-analysis
Source: PLoS One. 2022 May 6;17(5):e0265411. doi: 10.1371/journal.pone.0265411 (PMC9075620; doi:10.1371/journal.pone.0265411)
Supplement: Attachment — Submitted filename: SR_KAP_Ethiopia_reviewAA.docx [file pone.0265411.s004.docx]

Reviewer (Asri Adisasmita) Comments to Authors:

Thank you for the opportunity to review your valuable research.

1. Abstract:

Since this is a Systematic Review (SR) article, it is advisable to follow the guideline in reporting a SR as listed in PRISMA Guideline. It seems that the abstract did not contain information about inclusion and exclusion criteria, methods to assess risk of bias, a brief summary of limitations, register name and registration number. It would be more complete if the abstract could contain the above information that are lacking.

2. Introduction:

The introduction was well-written.

3. Methods:

The methods section was, in general, written well. Some missing information could be added:

a. The search strategy was listed only for Pubmed, but not mentioned for the other databases. Could that be also provided in the article?
b. For Selection Process and Data Collection Process, if automation tool was not used, please add information as such.
c. The use of grey literature was not mentioned in the method section, however, it was mentioned in section on Strength and Limitation of the Study (third line) that one the strength of this study is …”the accessing of grey literature’s”. Could you explain this?

4. Results:

The results section was also well-written, however, there is some a question:

a. Sensitivity analysis, it is written as such: “A leave-out-one sensitivity analysis was done to identify the effect of each study on the pooled prevalence of good level of knowledge, positive level of attitude, and poor level of practice of kangaroo mother care of postnatal women by excluding each study step by step. The result showed that the excluded study brings significant change to the overall prevalence of a good level of knowledge, positive attitude, and poor practice.” Based on Table 1, Good Knowledge was supported by 5 studies, Good Attitude by 5 studies, and Good Practice by 16 studies, and from the Forest Plots (Fig. 2, Fig. 3, and Fig. 4), the number of studies listed in Table 1 and the corresponding figures of forest plot were exactly the sam. There was no exclusion of any studies. The facts that there were only 5 studies for Good Knowledge and Good Attitude, is because the number of studies that provided information for the two outcomes were only five. There was no exclusion of any study as claimed above. Could the authors clarify this matter?
b. How to explain the connection between data estimates listed on Table 2 and those in figures of forest plot (Figs. 2, 3, and 4).

5. Discussion:

In the discussion section, the findings from this SR was compared to findings from national surveys done in developed countries (Denmark, America – meaning USA?, Taiwan, and Canada). Would the comparison be comparable? First, national survey data form the mentioned developed countries usually includes large sample sizes, whereas the studies included in this SR involved relatively small number of respondents. Secondly, eleven of 16 studies were from hospitals, whereas national surveys would include respondents from the community. Thirdly, would comparing KMC provided by mothers from Ethiopia to those from the above mentioned countries be a suitable comparisons? Especially if we consider the differences based on social, economic, cultural and daily needs/problems faced by the mothers. It would be more meaningful to compare the findings with those from countries in Africa or developing countries in Asia, as well as in Latin America.

Thank you. I hope that this Systematic Review will add significant knowledge about KMC implementation.
