# Supplementary material for: Knowledge, attitude and practice towards kangaroo mother care among postnatal women in Ethiopia: Systematic review and meta-analysis
Source: PLoS One. 2022 May 6;17(5):e0265411. doi: 10.1371/journal.pone.0265411 (PMC9075620; doi:10.1371/journal.pone.0265411)
Supplement: Attachment — Submitted filename: Rebuttal letter.docx [file pone.0265411.s005.docx]

Rebuttal letter

| Comments in each section | Reviewers comment | Authors response |
| --- | --- | --- |
| **General comments**   - Spelling and English language editing requirement - Usage of plos one format style | Reviewer 1 | - The whole document was intensively edited and modified for English language and spellings - The document revised by English grammarily online editor and <https://www.scribendi.com/> online editor extensively |
| **Abstracts** |  |  |
| **Key word**   - The word’ Emphyisotomy” does it typing error**?** | Reviewer 1 | Yes it was a typing error and the whole **key words** are: knowledge, Attitude, Practice, kangaroo mother care, Ethiopia |
| -no inclusion and exclusion of studies  -no brief summary limitations  -risk of bias assessment  -registration name and number | Reviewer 2 | - Out of 273 articles retrieved, 16 studies met eligibility criteria and included in this study  -the Joanna Briggs Institute tool was used to assess risk of bias.  -This study is limited with postnatal women and not representative of their husbands and health providers  - This systematic review and meta-analysis study was not registered under Prospero, but we checked that any author has registered it yet. |
| **Methods** |  |  |
| **Eligibility criteria**   - Time frame should be mention for data accessing and search of literatures - Explain Grey literatures - Specify about funding | Reviewer 1 | - The electronic search of literatures was done from May 30, 2021 to June 30, 2021, and all available studies published in English from inception up to June 30, 2021 were used. - Grey literatures are unpublished documents of organizational reports, scientific research papers, different papers reporting results of conference, workshops on found from their webpages. - These grey literatures reduce publication bias and increase reviews comprehensiveness this could be taken as strength of study. - The authors receive no specific funding. |
| - **Operational definition** |  |  |
| Practice was the study participants who responded more than 50% responded questions were categorized as having a good ATTITUDE (???). Please check. | Reviewer 1 | It was typing error and corrected as “Those study participants who responded to ≥50% of practice-related questions were categorized as having a good practice whereas those respondents who answered <50% of questions were considered as having poor practice”. |
|  |  |  |
| **-**the **s**trategy presented for PubMed only how other data bases were searched? | Reviewer2 | In manual search, We apply different to make manual searching: searching references from included studies/reviews, contacting authors and experts, performing what is known as citation tracking in which the reviewers track all the articles that cite each one of the included articles, and this might involve electronic searching of databases; and thirdly, similar to the citation tracking, we follow all “related to” or “similar” articles.  **In google scholar**:  Where my words occur: in the title of the article: 1. With all of the words: knowledge ,attitude and practice of kangaroo mother care With at least one of the words: kangaroo mother, kangaroo mother care, kangaroo mother care method  2. With all of the words: skin to skin contact With at least one of the words: skin to skin contact, skin to skin care practice.  In EMBASE those friends who attend their education in abroad accessed the searching also participated and we used Boolean operators with the quick search and Emtree terms (a controlled vocabulary or standard words used to make searching easier) to get the available articles in the database whereas in web of science we used synonyms, Boolean operators, key words, and topics were used. In Google scholar and manual search a combination of the above key terms/phrases (“Knowledge of kangaroo mother care”, “attitude of kangaroo mother care”, “kangaroo mother care”, “low birth weight neonate”, “preterm neonate”, “newborn care practice”, “kangaroo mother”, “skin to skin care practice”, “skin to skincare”, “kangaroo mother care method, and Ethiopia) were used. |
| The search strategy in the Pub Med : ((((( Knowledge, please check is it typo error? | Reviewer 1 | It was a typing error. |
| Grey literature | Reviewer 2 | The usage of grey literature was not mentioned due to typing error from the methodology part. Grey literatures are unpublished documents of organizational reports, scientific research papers, different papers reporting results of conference, workshops on found from their webpages.  These grey literatures reduce publication bias and increase reviews comprehensiveness this could be taken as strength of study. |
| **Study selection and data extraction** |  |  |
| Any ideal difference that happened due to extraction was also selected by the fourth articles. Please explain what you meant is by the four persons research team member? | Reviewer 1 | It was a typing error and corrected as “Any ideal difference that happened during extraction was also solved through discussion lead by the fourth author”. |
| 3.data availability issue | Reviewer 2 | All relevant data are within the Manuscript and its Supporting Information files |
| **Results** |  |  |
| a. Sensitivity analysis needs   Clarification with the forest plot and the number of studies in the analysis | Reviewer 2 | Among the total of 16 studies, only 5 studies provided information for Good Knowledge and Good Attitude, but all 16 studies reporting practice. The number of studies listed in Table 1 and the corresponding figures of forest plot were exactly the same. There was no exclusion of any studies. **This was why when we computed sensitivity analysis each study was omitted but after omitting a single study the result obtained from the remaining study represents the study that we omitted from the analysis. The same was to each study for all 5 studies reported knowledge and 5 studies reported attitude.**  **That was the same for all studies reported practice. In the analysis we omitted each study one by one, finally the result was presented with each omitted study and the estimated value. For example: in case of articles reported about when we omitted “ mose et.al” and calculated sensitivity analysis for the remaining 4 articles we found 72.22.**  **The same method we followed and obtained results for the rest 4 authors. This was the same for attitude and practice. Therefore, the number of studies was not reduced in final report, but in analysis.**  **In addition to this, the result from forest plot used to check the presence of asymmetry for showing heterogeneity, but sensitivity analysis for the effect t of each single study on overall.** |
| b. How to explain the connection between data estimates listed on Table 2 and those in figures of forest plot (Figs. 2, 3, and 4). | Reviewer 2 | In table2: the pooled estimate of each of knowledge, attitude and practiced was computed by using sensitivity analysis. In this case, the pooled estimate was calculated by omitting each study turn by turn.  In the forest plot (figs.2,3,4), it is the graphical display of the observed effect , confidence interval and the weight of each study.  Therefore, the connection between them was both of the two showed estimated values of knowledge, attitude and practice. In forest plot, the estimation was by the entire studies, but by sensitivity analysis by omission of each study. |
| **Discussion** |  |  |
| Comparison of the findings with developed countries of (America, Canada, and Taiwan) not appropriate. Compare. | Reviewer 2 | Comparing our finding with related studies done from developed countries was not appropriate. We have found that the points raised by you are convincible. We compared with findings from developing countries (Singapore and New Zealand). |
| **Disclosure** |  |  |
| - Funding issues of the study | Reviewer 1 | The author received no specific funding for this work. |
| **Reference** |  |  |
| “Alelign,Zewuditu/unpublished  Dawit , Aster /unpublished  Haftey Gebremedihn et,al /unpublished” which has currently not yet been accepted for publication. Please remove this from your References and amend this to state in the body of your manuscript: (ie ““Alelign, Zewuditu [unpublished]”) as detailed online in our guide for authors | Reviewer 1 | This was a typing error. While we say unpublished work we mean to say deposited articles (preprints) and master thesis. Accordingly, in plos guideline, We found that these preprints and master thesis can be list as other list of references’. Therefore, We adjust based on that. **For example in PLOS One reference guide line format regarding deposited articles (preprints,e-prints**) i.e= 1. Krick T, Shub DA, Verstraete N, Ferreiro DU, Alonso LG, Shub M, et al. Amino acid metabolism conflicts with protein diversity. arXiv:1403.3301v1 [Preprint]. 2014 [cited 2014 March 17]. Available from: <https://128.84.21.199/abs/1403.3301v1> **In case of master thesis or doctoral dissertation We found the sample in Plos-One reference guideline i.e** 2. Wells A. Exploring the development of the independent, electronic, scholarly journal. M.Sc. Thesis, The University of Sheffield. 1999. Available from: <http://cumincad.scix.net/cgi-bin/works/Show?2e09> |
| **General comments** |  |  |
| File format using plos one |  | The file adjusted based on plos-one format. |
| Figure should use PACE |  | The figures are corrected by using **PACE** to make suitable for plos. |
| Risk of bias assessment |  | Risk of bias assessment to check reliability and validity of data extraction tool were assessed and send as supplementary file |
| Quality assessment |  | The quality assessment score for each study using Jonna Brigg’s institute also reported as a supplementary file. |
| Language track changes |  | The entire manuscripts showing language track changes reported as a supporting information |
